# The Role of Oxytocin and Vasopressin in People with Borderline Personality Disorder: A Closer Look at Adolescents

**DOI:** 10.3390/ijms252212046

**Published:** 2024-11-09

**Authors:** Magdalena Uzar, Monika Dmitrzak-Węglarz, Agnieszka Słopień

**Affiliations:** 1 Department of Child and Adolescent Psychiatry, Karol Jonscher Clinical Hospital, Poznan University of Medical Sciences, Szpitalna St. 27/33, 60-572 Poznan, Poland; 90379@student.ump.edu.pl; 2 Department of Psychiatric Genetics, Medical Biology Center, Poznan University of Medical Sciences, Rokietnicka St. 8, 60-806 Poznan, Poland; mweglarz@ump.edu.pl

**Keywords:** borderline personality disorder, adolescents, oxytocin, vasopressin

## Abstract

Borderline personality disorder constitutes a significant medical challenge. Despite the fact that its occurrence among adolescents is currently attracting increasing interest from both clinicians and researchers, there is still insufficient data on this phenomenon. The etiology and maintenance of borderline personality disorder are not yet fully comprehended. Neuropeptides, including oxytocin and vasopressin, are considered to be involved in the development of this condition. The mechanism behind the actions of these neurohormones requires further investigation. Our work aims to collect and analyze the available research and existing hypotheses on the role of oxytocin and vasopressin in people with borderline personality disorder, with special attention drawn to adolescents suffering from this condition.

## 1. Introduction

Currently, it is recognized that borderline personality disorder (BPD) has a multifactorial etiology, including neurobiological factors such as, but not limited to, the commonly postulated influence of various neurohormones [1,2,3]. The authors decided to investigate oxytocin and vasopressin, considering them to be a few of these neurohormones which are presently regarded as potentially being most connected to the development of BPD [3,4].

Despite numerous studies on the role of oxytocin and vasopressin in many aspects of human social life, the mechanisms of their action have not yet been fully elucidated. During adolescence, various neurobiological, physiological, and psychological changes occur in the human body, which makes it a unique period of development [5,6]. There is evidence that these changes concern, among others, the hypothalamic–pituitary–gonadal axis and the hypothalamic–pituitary–adrenal axis which are significantly related to the action of the neuropeptides mentioned above [7].

The available data indicate the occurrence of abnormalities in oxytocin and vasopressin expression in people with various mental disorders, including those with borderline personality disorder [3,8,9,10]. Nevertheless, there is a scarcity of research on these neurohormones in adolescents with BPD.

In order to summarize the current state of knowledge of oxytocin and vasopressin in people with borderline personality, we performed a detailed analysis of numerous studies. The following work first presents the available data on the effects of oxytocin and vasopressin in healthy people, then it synthesizes the knowledge of borderline personality disorder, including data on adolescents with this diagnosis, and finally, it focuses on oxytocin and vasopressin disturbances in people with BPD. We drew particular attention to the analysis of data on this topic regarding adolescents with borderline personality disorder.

## 2. Oxytocin

### 2.1. Introduction

Oxytocin is a neurohormone that is predominantly synthesized in magnocellular and parvocellular neurons in the paraventricular, supraoptic, and intermediate accessory nuclei of the hypothalamus of mammals, including humans, from where it is transferred to the posterior pituitary gland, and then is secreted to the bloodstream [11,12,13]. Moreover, there is evidence that magnocellular oxytocin neurons project collaterals from the hypothalamic–neurohypophysial tract to many regions of the forebrain, and that parvocellular oxytocin neurons project this collaterals to the midbrain, brainstem, and spinal cord [14]. In addition, oxytocin can also be synthesized in other areas of the body, such as the heart, ovaries, and adrenal cortex [11,15]. It is classified as a neuropeptide that acts as a hormone in peripheral blood and as a neurotransmitter in the central nervous system [16].

**Figure 1:**
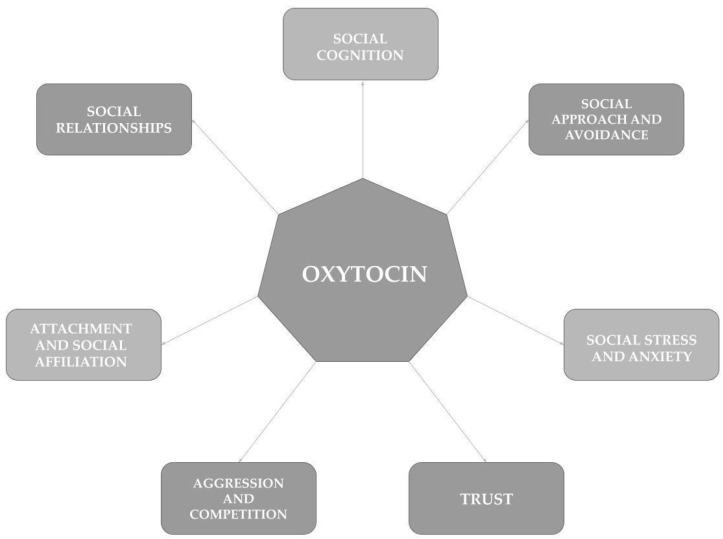
Aspects of social life that are influenced by oxytocin.

Oxytocin has many different functions, including reproduction, food intake modulation, pain perception, and the regulation of both the cardiovascular system and autonomic functions, such as breathing [14]. Moreover, as commonly known, oxytocin is called the “love hormone” due to its role in the relationship between a mother and a child and in romantic relations [17]. Oxytocin, however, plays a much broader role in human social life through modulating social cognition and behaviors related to social approach and avoidance, social affiliation, and attachment [12,16,17] (Figure 1).

### 2.2. The Influence of Oxytocin on a Child’s Relationship with Their Primary Caregiver and the Formation of Attachment

The function of oxytocin in a mother’s body is particularly essential since it causes uterine contractions during childbirth [18], affects a woman’s physiological and psychological adaptation to the role of a mother by increasing sociability and reducing anxiety [19] and, last but not least, has a function in the secretion of milk during lactation [19,20]. It has been shown that in the early period after birth, breastfeeding leads to a more frequent secretion of oxytocin in the mother, which results in decreased levels of the adrenocorticotropic hormone and cortisol, reducing any anxiety she may feel and prompting her to feed her child [17,21,22,23].

Furthermore, some research indicates that oxytocin plays a role in the formation of attachment between a mother and her child, and it becomes the basis for the formation of other interpersonal relationships established and maintained by people throughout life [8,24]. Attachment is defined as a selective pattern of responses to other people [25]. This term was first coined by John Bowlby, who stated that all people need to create close emotional bonds with others in order to experience a sense of security and to regulate their emotions [26,27]. A healthily developing infant exhibits attachment behaviors such as seeking closeness, smiling, and clinging. If an adult responds to these behaviors with touching, hugging, and using a calming tone of voice, these behaviors on the part of a child towards the caregiver become more intense, and, as a result, an attachment is created [26,28]. Available evidence demonstrates the potential role of oxytocin in higher-level attachment behaviors, such as affectionate verbalizations towards infants and the mirroring of children’s behavior by parents [16,29]. Moreover, the intranasal administration of oxytocin in fathers results in an improvement in the quality of interactions with their toddlers–they more frequently encourage their children to explore and they express less hostility [30]. As commonly believed, people learn to regulate their emotions through the repeated experience of their internal states being understood and responded to correctly by their primary caregiver [26]. Over time, a child’s specific experiences in the relationship with their caregiver are transformed into a system of internal representations called “internal working models” [26,31]. Notably, the relationship between oxytocin and parental care appears to have some genetic basis. The *CD38* (CD38 molecule) gene, for instance, encodes a protein that plays a role in the release of oxytocin by hypothalamic neurons, while the *OXTR* (oxytocin receptor) gene encodes the receptor for this neurohormone [32,33]. Researchers have found that polymorphisms in genes such as *CD38* (rs3796863) and *OXTR* (rs2254298 and rs1042778) are associated with reduced plasma oxytocin levels [34]. The authors demonstrated that both oxytocin levels and the above-mentioned genes’ variations are correlated with how parents interact with their children—parents with low plasma oxytocin touched their children less often, similarly to parents with *CD38* risk alleles and those with *OXTR* risk alleles [31]. It has also been found that mothers with the AA or AG variants of *OXTR* rs53576 showed less sensitivity towards their children than mothers with the GG variant [35].

The formation of a child’s relationship with their caregiver, during which the child experiences adequate care and the parent correctly recognizes and responds to the child’s internal states, leads to the development of a secure attachment style in the child. Abnormalities in a parent–child relationship may result in the development of non-secure attachment styles [26]. Additionally, the current state of knowledge on the topic seems to recognize other factors that can influence the formation of attachment, including neurobiological factors, the family system, and the broader sociocultural environment [36,37]. Interestingly, the available data indicate some connections between oxytocin and attachment. A study on one hundred and seventy-six infants aged 12 to 16 months showed a relationship between attachment security and *OXTR* gene polymorphism, demonstrating a significant correlation between the A allele of *OXTR* rs2254298 and attachment security in “non-Caucasian” infants, but, curiously, not in “Caucasian” ones [38]. In addition to indicating potential genetic determinants of attachment development, the study results also suggest the occurrence of possible differences between humans of various ethnic origins in this respect [38]. Furthermore, it is worth mentioning that connections between oxytocin and attachment are also visible in adulthood. A study by Plasencia et al. proved that low plasma levels of oxytocin are associated with high attachment anxiety [39]. Notably, the longitudinal administration of oxytocin (for two weeks) in healthy men leads to reductions in attachment avoidance, i.e., a lower reluctance to closeness and trust towards others [40]. Moreover, Bart et al. found that a single intranasal administration of oxytocin significantly affected the way healthy men perceived relationships with their mothers: the participants with more secure attachment recalled their mothers as more caring and close during childhood after the administration of this neurohormone, while those with a more anxious attachment recalled them as less caring and close [41]. According to some authors, these data indicate that the effects of oxytocin may strengthen both positive and negative interpersonal schemas [24].

The available literature evinces that a parent’s attachment style may indirectly influence the development of their child’s attachment [42]. Firstly, research indicates that higher levels of attachment avoidance and anxiety in mothers are correlated with reduced quality in the relationship with their children [43]. Secondly, the meta-analysis of Koehn and Kerns reported an association between attachment in children and the quality of parenting care—parents of children with more secure attachment are more responsive, strongly support the autonomy of their children, and use milder behavioral control strategies [44]. Research implies the potential involvement of oxytocin in these processes. There is evidence for higher levels of oxytocin in mothers with a secure attachment compared to mothers with insecure attachment styles during interactions with their several-month-old children [9,45].

Creating a primary bond with a caregiver is a foundation for the child’s further development. However, the available evidence suggests that throughout life, a person may attach to different people in various ways, and, as has already been mentioned, there are many factors that need to be considered when analyzing the formation of attachments [31,36]. Nevertheless, the quality of primary relationships has a significant impact on emotional and social functioning in the later stages of life. This influence is particularly expressed in the connection between the child’s relationship with their primary caregiver and the development of their ability to mentalize [28,37]. Mentalization is a higher-order mental capacity that allows people to understand their own and other people’s internal states [28,37]. It is a form of social cognition, and it overlaps in meaning with terms like, for example, Theory of Mind or empathy [46,47,48]. The potential effect of oxytocin on social cognition, including mentalization, is presented in the following section.

### 2.3. The Effect of Oxytocin on Social Cognition and Behaviors

The literature postulates the involvement of oxytocin in processes related to social cognition, as well as social behaviors.

#### 2.3.1. Social Cognition

Some studies have suggested the positive influence of intranasal oxytocin administration on mentalizing and a general ability to empathize [49,50,51,52]. There is also some evidence delivered by genetic research. Wu and Su demonstrated that children aged 3 to 5 who are GG carriers of *OXTR* rs53576 have increased abilities connected with Theory of Mind in comparison to children with the AA genotype [53]. Similar data are available for the empathic capacities of adults with the GG genotype [54]. On the other hand, a meta-analysis created by Leppanen et al. showed no apparent effect of intranasal oxytocin administration on the emotional Theory of Mind in healthy people [55]. Thus, it is currently alleged that its mechanism of action can be more complex and selective, and does not cause generalized improvement in social cognition abilities.

Notably, the impact of oxytocin on the cognitive aspect of empathy has been pinpointed [56]. Oxytocin mediates the perception and recognition of social signals, especially facial expressions [57,58,59,60,61]. A study by Nishizato et al. demonstrated that in a group of infants and children (5–90 months of age) there is a simultaneous increase in attention drawn to non-social cues and a decrease in both visual attention paid to social cues and levels of oxytocin in the saliva with age [62]. The possible influence of oxytocin on processing information related to the eye area has been emphasized. Firstly, there are data indicating that oxytocin modulates the eye gaze by increasing gaze focus on the eye area in the case of faces with neutral and positive expressions, as well as by decreasing eye focus on faces expressing anger [63,64,65]. Secondly, the study by Nishizato, previously mentioned, discovered a correlation between the attention drawn to the eye area and salivary oxytocin levels, as well as relationships between visual attention paid to the eye area, *OXTR* gene polymorphism, and age—children over 24 months of age with the AA genotype reduced their fixation span on this area in comparison to those with the GG genotype [62]. Furthermore, it has been shown that in adults, oxytocin elevates their ability to correctly assign people’s internal states on the basis of signals related to the eye area, as assessed by the Reading the Mind in the Eyes Test—RMET [49]. However, research indicates that in this test, this improvement is visible only for difficult items and not for simple ones [49]. Moreover, the previously mentioned meta-analysis demonstrated that oxytocin significantly enhances the recognition of basic emotions, particularly fear, in healthy people [55].

The relationship between oxytocin and the ability to empathize may depend on many factors. A study by Burkhouse et al. demonstrated that children who are G homozygotes of *OXTR* rs53576 and whose mothers were depressed during children’s lives exhibit higher sensitivity in the recognition of sad faces and lower sensitivity in detecting happy ones in comparison to children of mothers without depression. This association was not visible among carriers of the A allele of this gene [66]. Furthermore, the meta-analysis by Chander et al. indicated the possible impact of age and ethnicity on this relationship. It has been demonstrated that there are significant differences between GG and A allele carriers in a general ability to empathize, but only when considering the above-mentioned factors. The authors show that G homozygotes have a better general capacity to empathize than carriers of the A allele only in a group of young to middle-aged adults (not in the group of older ones) [67]. Moreover, they imply that G homozygotes have a higher cognitive empathy ability if they are of Asian ethnicity and an augmented affective empathy capacity if they are Europeans [67].

The study by Hurlemana et al. is also worth emphasizing. The authors found that while the intranasal administration of oxytocin enhances socially reinforced learning in healthy men, it has no effect on learning outside of the social context [51,58]. These results seem to be particularly interesting when we analyze them in the light of the current concept of associations between mentalizing, epistemic trust, and social learning [37].

#### 2.3.2. Social Stress and Anxiety

Studies have shown that social stress causes an increase in oxytocin in the blood and saliva of healthy adults [68,69,70], as well as in the saliva of healthy adolescents [70]. There is also evidence that since oxytocin may influence the limbic system, it reduces anxiety and the neurobiological/neuroendocrine response to stress in social situations, including the effect on the hypothalamic–pituitary–adrenal (HPA) axis [17,56]. On one hand, it has been postulated that the impact of oxytocin on the HPA axis can mediate both the alleviation in responses to threat and a decrease in aggressive behaviors by reducing cortisol secretion, and, consequently, leading to stress reduction [71]. On the other hand, there are some studies demonstrating contradictory results, including research by Tabak et al. which indicates that elevated mean peripheral oxytocin reactivity after a relational laboratory task (intended to induce an emotional state regarding relational transgressions) is connected with increased post-conflict anxiety. However, there was no association between these variables and task-related changes in the cortisol level [72]. Moreover, a meta-analysis by Brown et al. shows a positive correlation between oxytocin and cortisol levels after novel or stressful situations [73], which may indicate different relationships between these hormonal systems. An interesting perspective was also shed by research by Bernhard et al. which showed that psychosocial stress in adolescents leads to an augmentation in the level of oxytocin and then, after a few minutes, raises the level of cortisol (both measured in the saliva) [70]. These results mirror the outcomes of studies in adult populations [68,69]. Furthermore, Doom et al. found differences between forms of social support and levels of oxytocin under stress in boys (both children 9–10 years old and adolescents 15–16 years old). Boys who prepared for a speech with a parent exhibited elevated levels of oxytocin in the urine than those who were helped by a friend [74]. In addition, cortisol levels were increased if adolescents worked with friends, but not when they prepared with parents. Interestingly, this difference was not observable in a group of children [74]. The above results indicate potential changes in the oxytocinergic system that occur with age.

Moreover, it has been demonstrated that in adults, during the second stress phase, higher oxytocin secretion is correlated with faster vagal recovery [69].

Based on the above-mentioned research, it has been suggested that the anxiolytic and stress-reducing effects of oxytocin may be related to its earlier release and that the subsequent effect on other stress hormones, including cortisol, has “a recovery-boosting rather than a reactivity-buffering effect“ [70]. Furthermore, it has been pointed out that the correct interpretation of such divergent results may only be possible after taking into account other characteristics, especially individual or contextual factors [73,75]. One of the foundations for this hypothesis is the research by Meinlschmidt et al. according to which the intranasal administration of oxytocin to men who experienced early separation from their parents resulted in a smaller decrease in cortisol levels than in the group of men who did not experience separation [76]. The study by Tops et al., provides another argument, according to which state and trait anxiety and cortisol levels positively predict oxytocin levels, and the relationship between oxytocin and anxiety is modulated by attachment, understood as the tendency to express and share internal states with friends [77,78].

#### 2.3.3. Trust

Oxytocin seems to play a complex role in trust-based behaviors [17,79,80]. Trust is perceived to be a precondition to social approach and social affiliation [81]. The available research demonstrates that the intranasal administration of oxytocin leads to raised trust within social situations [81,82], including after the experience of having been betrayed in a relationship [56,83]. However, there are also contradictory results—Declerck et al. found that the intranasal administration of oxytocin does not increase trust in a minimal social contact situation [84]. Moreover, there is evidence that oxytocin can elevate feelings of mistrust in situations lacking social information, which can result in reduced cooperation [84,85]. Interestingly, the meta-analysis by Van IJzendoorn and Bakermans-Kranenburg demonstrated that the intranasal administration of oxytocin increases the level of in-group trust, but it has no effect on the level of out-group trust [61].

Furthermore, associations between oxytocin, trust, and aggression have been postulated. A study conducted on a group of healthy men aged 18 to 35 showed that low levels of interpersonal trust combined with low levels of oxytocin in the urine are associated with a lifetime history of aggressive behaviors [71].

#### 2.3.4. Social Approach and Avoidance

Zik J. et al. hypothesize that oxytocin may perform a function of a mediator both in the processes of social approach and in defensive situations [16]. These authors assume that first, it evolved from an ancient nonapeptide that enabled invertebrates to maintain boundaries between the organism and its environment, and secondly, with the cognitive and social development of humans, the function of oxytocin changed from keeping physical boundaries to maintaining and regulating the maintenance of social boundaries [16,86].

Oxytocin seems to engage in deeming situations and relationships as threatening or secure. The hypothesis that this neuropeptide modulates threat sensitivity and avoidance in healthy people is supported by many studies [81,87]. The meta-analysis by Leppanen et al. demonstrated that a single intranasal administration of oxytocin intensifies the startle response to threats in a group of healthy people [88]. Interesting results are also presented by studies on approach–avoidance tasks. Healthy individuals respond by approaching when seeing happy faces and by avoiding contact when seeing aversive or threatening stimuli, such as angry faces. For this reason, these people respond faster to approaching a happy face and avoiding an angry face than the other way round—the so-called congruency effect [87,89]. It has been shown that the intranasal administration of oxytocin in healthy men with low levels of social anxiety leads to an enhanced approach towards angry faces [87,90]. Other researchers, however, indicated that the intranasal administration of this neuropeptide in women reinforces their approach to pleasant social stimuli [87,91].

Interestingly, some evidence suggests that oxytocin strengthens the tendency to share emotions socially [58,92]. However, the meta-analysis by Leppanen et al., mentioned in the previous section, showed that a single intranasal administration of oxytocin causes an increase in the expression of positive emotions only [55].

Studies indicate that due to the modulation of approach and avoidance behaviors, oxytocin significantly contributes to the regulation of intra- and inter-group processes and behaviors. The available evidence shows that the tendency for intergroup discrimination, i.e., treating people belonging to a given group differently than people outside it, results primarily from in-group love, not out-group hate [93,94]. Curiously, research demonstrates that oxytocin strengthens intergroup discrimination [82,93], which is based on the intensification of in-group love by this neuropeptide [95]. Moreover, it has been proven that oxytocin motivates intragroup compliance with norms and rules, namely group conformity [93,96,97], as well as intra-group cooperation [93,98]. The above-mentioned results have been partially confirmed in the meta-analysis by Yang et al., which demonstrated that the intranasal administration of oxytocin significantly improves cooperation in interactive games based on trust, reciprocity, and resource management in in-group situations, but not in out-group situations. However, this association was significant only for men [99]. Furthermore, this effect was visible only in one-shot games, but not in repeated ones. The authors postulated that oxytocin increases “social acuity” of the players, reinforcing a “Tit-for-Tat strategy” [99]. Oxytocin was observed to promote cooperation during the first round, whereas in the consecutive games, the participants were seen to adapt their strategy to their opponents’ behavior [99].

#### 2.3.5. Aggression and Competition

There is evidence indicating the potential function of oxytocin in aggressive behaviors. Research by Fetissov et al. demonstrates that adults with aggressive tendencies have an increased level of IgM autoantibodies reacting with oxytocin [78,100]. Furthermore, it has been found that intranasal oxytocin attenuates aggressive tendencies in women with a high state of anxiety to the level of aggression in women with a low state of anxiety [7,101]. Remarkably, this effect was not visible among males [7,102]. Moreover, studies have shown that oxytocin prepares people for defensive aggression used to defend a group against an external threat [93,103].

There is a paucity of research on the relationship between aggression and oxytocin in adolescents. Hovey et al., have found that in teenage boys, single-nucleotide polymorphisms (SNPs) in the *OXTR* gene (*OXTR* s7632287 AA) were strongly associated with overt aggression [104]. Furthermore, Zhang et al. reported that adolescents with the TT genotype of *OXTR* rs237885 exhibit an increased risk of displaying aggressive behaviors compared to those with GG and GT genotypes [105]. In addition, the authors showed an interactive effect between the above-mentioned SNPs and the experience of physical abuse during childhood in these adolescents [105]. There was also some evidence for associations between severe, persistent, and pervasive aggressive behaviors in a group of 6–16 year-olds and single nucleotide polymorphisms in *OXTR* rs1042778 and rs6770632 [106]. This connection was gender-dependent—the first of the above-mentioned SNPs was correlated with aggression in boys, while the latter one was correlated with aggression in girls [106]. Shao et al. presented associations between yet another SNP, namely in *OXTR* rs53576, aggression, and life stressors in adolescents. It was revealed that there was a positive relationship between stressful events during a 12-month period prior to the study and a level of physical aggression and hostility among adolescents with the AA genotype, but not among those with the G allele [107].

Notably, there are also some interrelationships between oxytocin and feelings connected with competition, which clearly demonstrates that oxytocin can sometimes show its “dark side” as well. The available data indicate that while playing a game for money and after administering oxytocin, people who won a smaller amount than others show elevated jealousy, while those who won a larger amount exhibit increased “schadenfreude”, understood as joy at someone else’s failure [85].

#### 2.3.6. Oxytocin and Romantic Relationships

The available data point to the existence of links between oxytocin and the quality of romantic relationships. There is evidence that people who experience greater partner support have higher levels of oxytocin in the plasma, both initially and after experiencing a brief episode of warm contact with the partner [108]. Moreover, the intranasal administration of oxytocin not only enhances positive communication during flirtation between a woman and a man [16,109], but also reduces cortisol levels during couple conflicts [58,109]. On the other hand, there are scientific grounds to believe that the intranasal administration of oxytocin can elevate the propensity for aggressive behaviors toward a romantic partner in people with a tendency for physical aggression [110].

There are several models explaining the role of oxytocin in romantic relationships. The “Calm and Connect” model assumes that the level of oxytocin is positively correlated with the quality of relationships, which results from the properties of oxytocin, such as reducing anxiety and defensiveness, as well as strengthening behaviors aimed at maintaining close contact and intimacy [111,112,113]. Contrarily, according to the “Tend and Befriend” model, there is a negative connection between oxytocin and the quality of romantic relations, which is based on the assumption that an increase in oxytocin levels caused by difficulties in a given relationship will lead to a greater motivation to seek affiliation with other people [111,112,114]. Nonetheless, the available data do not allow for the unambiguous confirmation of any of these models [111,112].

Grebe et al. proposed the “Identify and Invest” hypothesis which draws from both of the above-presented approaches, assuming that a rise in oxytocin levels may occur both in connection with a strong sense of attachment to a partner and in a response to adverse events in a relationship, which lead to feelings of disengagement [111]. According to this thesis, the level of oxytocin is high at the beginning of a relationship due to uncertainty felt by partners regarding the status of their relationship, while it decreases with the duration and perceived stability of the relationship. The above postulates were confirmed by the research conducted by the authors [111].

The results of genetic studies on the connections between oxytocin and romantic relationships are also worth mentioning. The data on single nucleotide polymorphism in *CD38* rs3796863 indicate that individuals with CC (vs. AC/AA) genotypes present higher levels of communal behavior in their daily interactions with their romantic partners and elevated levels of relationship adjustment [115]. If the outcomes of the above study are compared to the previously mentioned study on the quality of parenting, according to which parents with the CC genotype of rs3796863 touched their children less often than those with the AA/AC genotypes, the potentially complex and ambiguous influence of oxytocin on interpersonal relationships becomes more visible [34,115].

#### 2.3.7. Neurobiological Basis for Social Functions of Oxytocin

A possible mechanism behind oxytocin facilitating the perception and recognition of relevant social stimuli, which leads to better social adjustment, is considered to both modulate neuronal activity in many areas of the brain involved in the processes responsible for quick social decision-making and to enable coordination between these regions (“interregional coordination”) [57,116]. Moreover, this neuropeptide is likely to affect processes related to social cognition, among others, by exerting different influences on distinct subregions of the amygdala. The authors suggested that the amygdala is involved in detecting and directing attention to social signals, especially those with negative meaning [16,117,118]. Oxytocin is speculated to affect dopaminergic processes in this area of the brain [16,117,118]. There are data that prove diminished activation in the lateral and dorsal regions of the amygdala in response to fearful faces and elevated activation while responding to happy faces [58,119]. Furthermore, oxytocin reduces the reactivity of the amygdala in reaction to masked emotions related to emotionally significant facial regions, such as the eye area in angry faces and the mouth area in happy faces [58,63]. In addition, there is evidence for a relationship between oxytocin-induced changes in social attention and strengthened connections between the amygdala and the superior colliculus [116,119,120].

Skyberg et al. performed a study involving children (5–11 year-olds) who observed animations which required them to make a judgment about internal states [121]. The above-mentioned research reports that increased levels of DNA methylation of the oxytocin receptor gene (*OXTR*m) are connected with amplified neural responses in the left temporoparietal junction (LTPJ) and inferior frontal gyrus (IFG) [121]. Both of these brain regions are considered to participate in processes related to mentalization [122]. Moreover, it has also been demonstrated that the social skills of these children seem to constitute a positive predictor of an increased activity in the LTPJ [121]. The above-mentioned results are similar to the outcomes of the study by Jack et al. in adults, which revealed that during the observation of animations, there is a significant relationship between the levels of *OXTR*m and brain activity in the regions involved in social perception and mentalizing (including the temporoparietal junction and the dorsal anterior cingulate cortex) [121,123].

On the other hand, there are also some contradictory data provided in different research, for example the study by Straccia et al., which demonstrated no effect of the intranasal administration of oxytocin on reaction time and accuracy while performing a mentalizing task, as well as on whole-brain neural activation and functional connectivity within the brain networks that are related to mentalizing [124].

Notably, it seems that the effect of oxytocin on the central nervous system, including the amygdala, is related to gender. It has been suggested that in healthy males, oxytocin reduces the activation of the amygdala in response to threatening stimuli and decreases the coupling of the amygdala to brainstem regions connected to autonomic and behavioral manifestations of fear [125,126]. On the other hand, there is also evidence that in men, oxytocin reduces amygdala responses to facial emotional expressions regardless of valence [126,127]. Contrarily, some research shows that intranasal oxytocin leads to the enhancement of amygdala reactivity to scenes depicting threats (both social and non-social) in females [128,129]. Interesting relationships confirming the above-described differences between the sexes in terms of the influence of oxytocin on neural networks associated with social cognition and behavior were also demonstrated by Feng et al., whose study concluded that while the intranasal administration of oxytocin in a situation of reciprocated cooperation causes an increase in activity in the caudate/putamen, the right frontal pole and the left medial part of superior medial prefrontal gyrus in men, it actually decreases in women [130]. The authors emphasized that the effect of oxytocin on the caudate may be related to the function of this area that is responsible for identifying social prediction errors which, in turn, enables one to make decisions about reciprocity by determining if the other person is trustworthy or not [130,131].

Moreover, the researchers suggested that the amygdala mediates the processes of social approach through dopamine-controlled oxytocin receptors in the central nucleus [16,117,118]. The study by Radke et al. pointed out that the intranasal administration of oxytocin reduces amygdala activity during threat approach but not during threat avoidance [132].

### 2.4. Factors Modulating Actions of Oxytocin

Firstly, when analyzing data from studies on oxytocin, it is necessary to take into consideration that the release of this neuropeptide is triggered by various processes like giving birth, breastfeeding, sexual activity, and stress [133]. Secondly, a lot of studies are based on peripheral levels of oxytocin and there is not enough data on the relationship between oxytocin levels in the central nervous system and in peripheral circulation [16,133,134]. However, a meta-analysis by Valstad et al. demonstrated that, on one hand, under baseline conditions, there is a lack of clear associations between peripheral oxytocin concentration and the level of this neuropeptide in the central nervous system, but on the other hand, there is a positive correlation between those levels in experimental contexts (after the intranasal administration of oxytocin and after inducing stress) [135]. Thus, it is recommended to be cautious to avoid drawing unambiguous conclusions.

Furthermore, numerous experimental studies are based on the intranasal administration of oxytocin. Researchers indicate that so far, it has not yet been fully explained how oxytocin administered in this way causes the observed social effects [136]. On one hand, there is evidence that this neurohormone is most likely transferred directly into the brain via olfactory and trigeminal nerve projections, while, on the other hand, it is indicated that it may also be transported to the peripheral blood through blood vessels in the nasal cavity and oral cavity, as well as partially entering the gastrointestinal system (after swallowing) and affecting the receptors present there [136,137]. Interestingly, as pointed out by some authors, it is likely that only a small amount of intranasally delivered oxytocin enters the brain directly, while the majority of it goes to the peripheral circulatory system. As a result, peripheral oxytocin levels may rise to supraphysiologic levels [138]. Moreover, it is postulated that the intranasal administration of oxytocin probably causes this neurohormone to accumulate in some parts of the central nervous system; hence, this method of administration does not lead to its more uniform action in different areas of the CNS [136]. Therefore, thoughtfully considering the aforementioned conditions while analyzing studies based on this method is advocated.

The available literature indicates that oxytocin levels and actions are age-dependent [62,67,74,139]. Doom et al. found that adolescents (15–16 years old) have lower levels of oxytocin in the urine before and after being exposed to stress in comparison to children (9–10 years old) [74].

Moreover, the relevant literature suggests that the effect of oxytocin appears to be conditioned by gender [58]. Firstly, plasma levels of oxytocin in women are higher than in men [39], which has also been demonstrated in childhood and adolescence [74]. Secondly, it has been shown that elevated levels of oxytocin in men are associated with reduced trait anxiety and lower levels of attachment anxiety, while increased levels of this neurohormone in women are associated with high levels of trait anxiety and augmented levels of attachment anxiety [39,140]. Furthermore, since oxytocin reduces the response of the amygdala to faces expressing anger and fear in men but increases it in women, it can be concluded that the effect of this neuropeptide on the amygdala as a modulator in the processes of detecting social signals may be gender-dependent [119,125,127].

Researchers indicate that the key to comprehending the role of oxytocin in human social behavior may be understanding the role of factors modulating its social effects in both healthy and mentally ill people [16,75]. Bartz proposed an “interactionist model” in which contextual and intrapsychic factors play a moderating role in the influence of oxytocin on social cognition and behavior [58,75].

Shamay-Tsoory and Abu-Akel presented a theoretical framework called the social salience hypothesis, according to which oxytocin increases the salience of social cues by its contribution to orienting attention to social cues through interaction with the dopaminergic system [141]. The authors emphasized that this hypothesis is rooted in works by Bart et al. and Olaff et al. [24,75,141] and they indicated that the available data show both the prosocial and non-social effects of oxytocin depending on the context, individual variables, and factors related to the object of a given activity [85]. Moreover, these researchers argue that oxytocin affects attentional orienting responses. To support this claim, they present data from studies showing, among others, more frequent gazes at the eye area under the influence of oxytocin and a rise in directing attention while reacting to emotional gaze cues [65,85,142]. Finally, they investigated the influence of the dopaminergic system on the detection of salient stimuli and its interaction with oxytocin [85].

## 3. Vasopressin

### 3.1. Introduction

Vasopressin, or precisely arginine vasopressin (AVP), similarly to oxytocin, is predominantly synthesized in the magnocellular and parvocellular cells of supraoptic and paraventricular nuclei in the hypothalamus and then transferred to the posterior pituitary gland, from which it is released to the bloodstream and various brain regions [14,143]. It acts as a hormone in the peripheral blood and as a neurotransmitter in the nervous system.

Arginine vasopressin is transported from parvocellular neurons to various brain regions, including limbic structures, such as the suprachiasmatic nucleus, the amygdala and hippocampus, and numerous autonomic centers in the brainstem (the nucleus tractus solitarius, the nucleus ambiguus, the rostral ventrolateral medulla, the phrenic nuclei, and the pre-Botzinger complex) as well as to the spinal cord [144].

**Figure 2:**
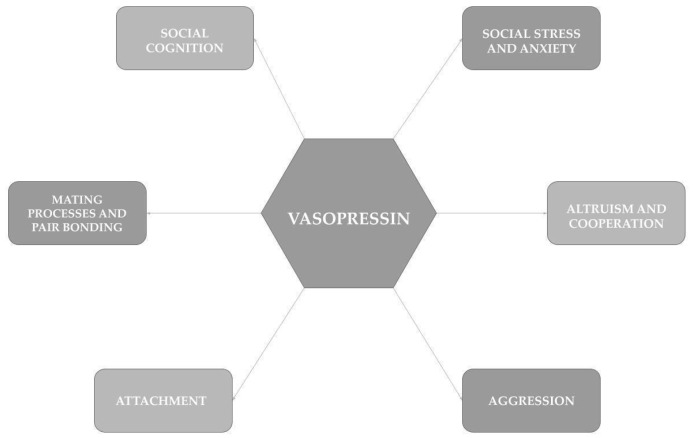
Aspects of social life that are influenced by vasopressin.

According to available knowledge, vasopressin is involved in numerous processes in the human body, including temperature regulation, as well as controlling cardiovascular and renal functions [145]. Although vasopressin is also postulated to participate in processes related to social cognition and social behavior, such as aggression, mating processes and pair bonding, as well as the formation of attachment between an infant and a parent, there is little research accessible on the dimensions of vasopressin action in humans [17,56,81] (Figure 2).

### 3.2. The Effect of Vasopressin on Social Cognition and Behaviors

#### 3.2.1. Social Cognition

There is a scarcity of research about vasopressin and social cognition in humans. A study by Tabak et al. indicates that vasopressin causes enhanced empathetic concerns in people who experiences more parental warmth from their fathers [146]. Notably, it does not lead to this effect in people who experienced higher levels of maternal affection [146]. However, there is also a study showing contradictory results. Uzasovsky et al. demonstrated that in men, the intranasal administration of arginine vasopressin (AVP) caused significantly worse emotion recognition in the Reading the Mind in the Eyes Test, but only in relation to the negative emotions of males (there was no effect on recognition of positive emotions and the emotions of females) [147]. Interestingly, Rilling et al., in their study involving both women and men who were asked to view faces, demonstrated that the intranasal administration of AVP caused distinct effects in women and men in relation to neural responses and to what they perceived as attractive [131]. In females, there was a significant increase in the perception of attractiveness of other women’s faces immediately after administration of AVP, while in males there was no direct effect. However, a few days after the administration, the positive rating of male faces in this group increased. Notably, there was no effect on the ratings of opposite-sex faces [131].

In the study by Thompson et al. it was demonstrated that in a group of healthy men, the intranasal administration of vasopressin had no effect on attention directed to facial expressions (emotionally neutral, happy, or angry) nor on the level of autonomic arousal that it caused. Nevertheless, it was observed to increase corrugator supercilii electromyogram (corrugator EMG) responses evoked by neutral facial expressions to the level of those elicited by angry faces [148]. The authors point out that the muscle group they studied is responsible for agonistic communication, which may indicate the impact of vasopressin on aggression in men, due to which they become more prone to respond to ambiguous social signals as if they were being threatened [148].

Furthermore, vasopressin seems to exert some influence on social memory. It has been revealed that in healthy men, the intranasal administration of arginine vasopressin enhanced their ability to remember both happy and angry faces more accurately, but not neutral ones, which possibly facilitates social behaviors connected with bonding and aggression [149].

Zink et al. demonstrated that after the administration of a placebo, the participants who were exposed to unfamiliar social stimuli exhibited a significantly higher level of activity in the left temporoparietal junction, while after the intranasal administration of vasopressin, the level of activity in this brain region was lower and comparable to that generated by exposure to socially familiar stimuli after the administration of a placebo [150]. Data from previous studies link this area of the brain with the Theory of Mind network [150,151]. According to the authors, the study results imply that vasopressin may trigger faster processing of unfamiliar social stimuli and lead to classifying them as familiar more promptly [150].

Research by Straccia et al., similarly to the data concerning oxytocin, showed no effect of vasopressin on reaction time and accuracy while performing a mentalizing task, as well as on whole-brain neural activation and functional connectivity within brain networks that are related to mentalizing [124].

#### 3.2.2. Social Stress and Anxiety

The available evidence indicates the involvement of vasopressin in the regulation of stress and anxiety in social situations, among others. Vasopressin appears to raise anxiety levels via augmented secretion in the hypothalamic paraventricular nucleus, leading to increased levels of anxiety both behaviorally and in neuroendocrine expression [17]. Interestingly, it has been demonstrated that high levels of vasopressin are connected with elevated attachment anxiety [39].

There is also evidence that the intranasal administration of AVP reduces state anxiety in men regardless of dose size; however, in people (both genders) carrying a “risk” allele in the RS3 promoter of the *AVPR1A* (arginine vasopressin receptor 1A) gene, this effect is visible also after administering lower doses [152]. For this reason, it is believed that carriers of this variant are more sensitive to the anxiolytic effects of arginine vasopressin. Moreover, women with the above-mentioned allele in the RS3 promoter exhibit lower levels of trait anxiety than women without this genotype [152].

In other research worth mentioning, after the intranasal administration of arginine vasopressin, participants were less willing to take a risk in a trial when the possibility of winning was high, which is similar to responses connected with elevated anxiety [153]. This effect was not observed in situations with a low probability of winning. Additionally, it was not dependent on social context and gender [153].

Notably, a significant connection has been found between the RS1 *AVPR1A* microsatellite variant and some personality traits like increased novelty seeking and decreased harm avoidance [56,154]. However, there is no association between these traits and the RS3 variant [154].

#### 3.2.3. Altruism and Cooperation

The intranasal administration of arginine vasopressin is alleged to increase risky cooperative behaviors in healthy adult males, which is, however, not connected with elevated general readiness to risky behavior or altruism [155]. Moreover, researchers demonstrated that during risky cooperative choices made by participants who received AVP, there is a visible down-regulation of activity in the left dorsolateral prefrontal cortex and increased functional connectivity between this region and the ventral pallidum. The authors emphasized that the former of these brain regions is associated with risk integration, while the latter potentially takes part in social reward processing [155,156,157].

On the other hand, the meta-analysis by Yang indicated that in healthy people, a single dose of intranasal arginine vasopressin administration weakens interpersonal cooperation in interactive games [99]. It is worth emphasizing that this influence of vasopressin was visible in repeated games but not in one-shot games, which may indicate that vasopressin reinforces social competition especially when it is necessary to become involved in interpersonal interactions repeatedly [99].

Interesting data were provided in a study by Feng et al., according to which the intranasal administration of arginine vasopressin caused women to lie more than those receiving a placebo, but also more than men under the influence of intranasal AVP [158]. This behavior was observed in situations that were regarded as beneficial for both the subject and other people. Moreover, the tendency of women who were receiving arginine vasopressin to be dishonest grew stronger over time. The authors indicate that the above results constitute an argument for the contribution of vasopressin to altruistic dishonesty [158].

Furthermore, it has been shown that there are potential relationships between the gene of the vasopressin receptor subtype (*AVPR1A*) and prosocial behaviors. Research has demonstrated that the length of the *AVPR1A* RS3 promoter region is positively associated with altruistic behaviors [17,159]. It has also been established that in a group of preschool children, there is a relationship between the *AVPR1A* RS3 327 bp allele and proclivity toward altruism—carriers of the 327 bp allele were more likely to exhibit altruistic behaviors towards strangers than non-carriers [160].

Moreover, there are potential associations between repeat lengths in the intron of *AVPR1a* and trust and reciprocity. Nishna et al. discovered that during a trust game, males with a short form of *AVPR1A* are more willing to send money to another player, even if there is a risk of a betrayal, than men with a longer form of *AVPR1A* [161]. In addition, the former group were more likely to return money to their opponent who had trusted them. However, attitudinal trust, understood as a general attitude to trusting other people, was not connected with the polymorphism of this gene [161].

#### 3.2.4. Aggression

There is also some data on correlations between vasopressin and defensive aggression. Kawada et al. performed an experiment with a preemptive strike game in which players needed to decide whether or not to use some of their resources to attack their opponent in advance in order to protect their own assets from being potentially harmed [162]. The researchers determined that the intranasal administration of arginine vasopressin increased the attack rate among adults, regardless of gender [162]. Interestingly, there was no connection between basal urinary arginine vasopressin levels and defensive aggression in this group [162]. Similarly, Berend et al. found no association between the level of vasopressin in the urine and levels of trust and aggression in healthy young men [71].

#### 3.2.5. Romantic Relationships

Associations between vasopressin and romantic relations have also been postulated. It has been shown that there are connections between the vasopressin receptor subtype (AVPR1A) gene and mating in men. Recurrent RS3 polymorphism significantly predicted performance on the Partner Bonding Scale [163]. It was also found that one of the alleles (334) of this gene is associated with the quality of marital relationships—its carriers reported a lower quality of marriage and experienced marital crises and threats of divorce more frequently. Simultaneously, their wives described lower marital satisfaction [17,163].

### 3.3. Factors Modulating Actions of Vasopressin

Firstly, when we analyze studies on vasopressin (as in the case of oxytocin), we must take into account the ways in which it is measured, including the fact that peripheral levels do not clearly reflect its levels in the central nervous system [136]. Secondly, in the case of experimental studies, the limitations associated with the intranasal administration of this neurohormone must be regarded [136].

Available research indicates some differences in the action of vasopressin depending on gender [131]. Moreover, it has been found that the level of vasopressin is age-dependent—it is higher in older adults (63–81 years old) than in young people (18–35 years old) [39]. However, there is a lack of research on the differences in the action of vasopressin in different age groups.

Similarly, like in the case of oxytocin, researchers emphasize that the discrepancies exposed in studies regarding the impact of vasopressin on social cognition and social behavior are probably both person- and context-dependent [158].

## 4. Interrelationships Between Oxytocin and Vasopressin

Owing to the fact that oxytocin and vasopressin are molecules with an analogous structure that are synthesized in similar areas of the human brain, their functional relationships seem to be worth delving into [14].

Oxytocin binds primarily to OXTRs, but also has some affinity for receptors for vasopressin–AVPR1A [164]. Moreover, it has been emphasized that both oxytocin and vasopressin influence various processes related to other hormonal systems as well as neurotransmitting pathways [39].

It has been found that in adults, levels of oxytocin and vasopressin are highly negatively intercorrelated, regardless of age and gender [39]. On one hand, researchers indicate that oxytocin and vasopressin have some opposing effects: arginine vasopressin is likely to increase alertness, arousal, anxiety, and activation, while oxytocin reduces anxiety, leading to relaxation [81,133]. Simultaneously, the available evidence, described in detail in the previous sections, has shown that actions of oxytocin and vasopressin are not always clearly delegated and should rather be perceived as more complex and occasionally overlapping, as well as dependent on other factors, including individual and contextual factors.

## 5. Borderline Personality Disorder

Borderline personality disorder (BPD) is characterized by affective instability, impulsivity, identity disturbances, and difficulties in interpersonal relationships [165]. People with this condition exhibit profound emotional dysregulation expressed in mood disturbances, chronic feelings of emptiness, self-aggressive behaviors, including repeated suicidal threats and suicide attempts, as well as aggressive behavior towards other people [166]. In addition, they are prone to experiencing transient paranoid ideations and dissociative symptoms while under stress [167,168,169,170]. Emotional dysregulation is closely connected with impulsivity, which is manifested in excessive money spending, risky sexual activity, substance abuse, reckless driving, and/or binge eating [166,171]. Moreover, one of the diagnostic criteria for BPD concerns identity disturbances which are characterized as a tendency for frequent changes in opinions, goals, values, career plans, sexual identity, and types of friends [172]. Identity problems are also expressed in difficulties in describing themselves and other people, as well as in exhibiting contradictory behaviors and beliefs. Furthermore, people with borderline personality disorder tend to perceive themselves in a negative way [172]. Last but not least, they often engage in chaotic relationships which trigger a strong fear of abandonment [173].

It is currently assumed that borderline personality disorder can be diagnosed in adolescence because the range of symptoms differs significantly from the turbulent emotionality and behavior typical for this period [174,175,176]. Early diagnosis allows prompt treatment, including, above all, appropriate psychotherapeutic interventions, which may be of great importance in preventing suicides of minor patients and improving their quality of life.

There are some differences between the symptoms of BPD among adolescents and among adults. The study by Zanarini et al. demonstrated that adolescents with borderline personality disorder, compared to adults with this condition, exhibit significantly lower levels of the following symptoms: quasi-psychotic thoughts, interpersonal feature issues (including dependency, masochism, devaluation, manipulation, and sadism), and countertransference problems [177].

The available data indicate that the disorder course pattern changes with age, ranging from symptoms related mainly to emotional dysregulation and impulsiveness in adolescence and early adulthood to a gradual decrease in the severity of the above-mentioned symptoms, with the simultaneous persistence of difficulties in interpersonal relationships and significantly impaired functioning in later stages of life. For instance, Jørgensen et al. showed that the course of the disorder in adolescents with BPD within 5 years of diagnosis is associated with a constant need for psychological support and pharmacotherapy in approximately half of cases, as well as with significantly deteriorated general functioning [178]. The above data suggest that the course of the disorder may be related to human maturation in a broad sense, which indicates the validity a requisite for searching for both psychosocial and neurobiological factors that may influence the development of BPD.

At present, it is recognized that borderline personality disorder has a complex, multifactorial etiology, and it develops as a result of multifaceted interrelationships between genetic, neurobiological, psychological, and environmental factors [4]. Nonetheless, the development of this disorder has not yet been fully elucidated.

There are no specific genes that are responsible for the development of BPD, but those that may contribute include the following: the serine incorporator 5 gene, tryptophan hydroxylase genes, the serotonin transporter gene, serotonin receptors genes, the monoamine oxidase A gene, dopaminergic genes, the catechol-O-methyltransferase gene, the tyrosine hydroxylase gene, the brain-derived neurotrophic factor gene, the *SCNA9* gene, the *AVPR1A* gene, and the neurexin 3 gene [179,180].

The available data indicate neurobiological differences in people with borderline personality disorder compared to the healthy population. Particular attention has been drawn to the amygdala, although research on this region in people with BPD has not yielded clear results. Some data suggest an increased concentration of total creatinine in the left amygdala, while others show a decrease in this concentration [181]. Furthermore, a reduction in the volume of regions such as the amygdala and hippocampus was found in people with BPD [181]. Interestingly, it was established that a reduction in the above-mentioned volumes, as well as in the frontal cortex, is associated with experiencing childhood trauma [182,183,184,185]. Lower gray matter volumes in regions like the bilateral middle temporal gyri, bilateral hippocampi, and right inferior frontal gyrus were also demonstrated [181]. On the other hand, patients with BPD have relatively greater volume in the right supplementary motor area, right cerebellum, and right middle frontal gyrus, including the dorsolateral PFC [181]. The results obtained from imaging studies in adolescents with BPD suggest that they exhibit cortical thickness in the default mode network and limbic-cortical circuit, as well as increased functional connectivity between the prefrontal cortex and both bilateral occipital lobes and the limbic system, but decreased connectivity between default mode network regions [186]. Uniquely, the above-described cortical thickness was positively correlated with emotional dysregulation [186].

There are also reports of the potential involvement of disturbances in neurotransmitters, including the above-mentioned neuropeptides, namely oxytocin and vasopressin, in the development and maintenance of this disorder [3,9]. Furthermore, researchers indicate a potential role of the hypothalamic–pituitary–adrenal axis in borderline personality disorder [187]. However, research on cortisol levels in people with BPD has not provided us with definite answers [181,187].

The history of patients with borderline personality shows frequent occurrences of adverse childhood experiences (ACEs), including, in a significant number of cases, violence [176,188]. The meta-analysis by Porter et al. demonstrated that the likelihood of disclosing childhood adversity is over thirteen times higher in people with BPD compared to healthy people [188]. It is worth emphasizing that although these people were found to experience all types of adversity (physical, sexual, and emotional abuse, as well as physical and emotional neglect) more frequently, a particularly strong effect was visible in the case of emotional abuse and neglect [188]. Moreover, the study by Temes et al. indicated that adolescents with BPD describe experiencing serious violence more often than healthy adolescents [189]. Adults with borderline personality disorder, on the other hand, reported both a more frequent occurrence of all forms of violence investigated in the study and a higher intensity of the maltreatment they experienced [189].

Researchers draw attention to prominent mentalization disturbances observed in people with borderline personality disorder, which are manifested both in a tendency for hypomentalizing, i.e., difficulties in understanding the internal states of others [4], and hypermentalizing, i.e., the excessive attribution of internal states to other people without objective evidence of their existence [37,190,191,192]. Characteristically, it seems that the propensity to hypermentalize becomes particularly visible in situations of strong emotions, especially in close attachment relationships, which contributes to the interpersonal difficulties experienced by patients with BPD. The latter of the above-mentioned predispositions has been observed both among adults and adolescents with this disorder [190]. However, as it has also been established in people with different mental illnesses, it can not be concluded as being specific to borderline personality disorder [190]. Moreover, there is evidence that both adults and adolescents with BPD present a lower capacity to infer about their own internal states [193]. Currently, it is alleged that people with borderline personality disorder do not exhibit a general deficiency in their ability to mentalize [4]. They seem to be characterized by a more complex pattern of mentalizing disturbances manifested both by hypo- and hypermentalizing [4].

There exists a high comorbidity between borderline personality disorder and other mental illnesses, including mood, anxiety, eating, and substance abuse disorders, as well as post-traumatic stress disorder and attention deficit hyperactivity disorder [194,195,196]. Moreover, similarities between borderline personality disorder and bipolar disorder deserve special consideration [197].

## 6. Oxytocin and Borderline Personality Disorder

### 6.1. Introduction

The research indicates that in adults with borderline personality disorder, firstly, the level of oxytocin in the plasma is lower than in healthy people [198,199,200,201,202,203,204], and secondly, the level of this neuropeptide in the plasma shows a negative correlation with the severity of the disorder [199,201]. However, there are studies that do not confirm both the first [205] and second correlations mentioned above [206]. It is worth emphasizing that significantly reduced oxytocin levels were also found in adolescents with this condition in comparison to healthy adolescents [201]. Moreover, adults with BPD present a reduced expression of the oxytocin receptor protein compared to the healthy population [198,204].

The available literature pinpoints possible associations between levels of oxytocin in patients with borderline personality disorder and adverse childhood experiences and social cognition and behaviors, including abandonment, trust, fear of compassion, and aggression. These relationships are described in detail in the following sections.

### 6.2. Relationship Between Oxytocin, Adverse Childhood Experiences, and Borderline Personality Disorder

The existing data demonstrate that the role of oxytocin in the development and maintenance of symptoms of borderline personality disorder may be closely related to adverse events in childhood, especially experiences of violence [201,207].

In the study of Mielke and colleagues, reduced levels of oxytocin in adult and adolescent females with BPD were associated with adverse childhood experiences [201]. In the above-mentioned research, ACE fully mediated the connection between borderline symptoms and the level of oxytocin in the plasma. The authors emphasized that these results can support the argument that oxytocin is one of the factors connecting childhood adversity and borderline personality disorder [201]. One of the arguments in favor of this connection is evidence included in a systematic review by Donadon et al., which shows a moderate association between having experienced early trauma and reduced endogenous levels of oxytocin, although there was no significant correlation between recent traumatic experiences and oxytocin [208]. Furthermore, there are also data demonstrating decreased oxytocin concentration in the central nervous systems of women who experienced childhood abuse and neglect [209]. However, no similar studies have been performed in the group of women with BPD.

It is worth emphasizing that research carried out on a group of adolescents established that those who have experienced violence but currently live in a stable environment exhibit a different pattern of oxytocin secretion throughout the day (measured in the saliva), i.e., it increased in this group in comparison to abused adolescents living in an unstable environment [208,210]. Moreover, in the latter group, there were significantly higher levels of cortisol after waking up than in the former group [210]. Notably, the above-mentioned associations of fluctuations in oxytocin levels and adverse childhood experiences have not yet been investigated in adolescents with BPD.

In addition to the above-mentioned results, it has been observed that among people with BPD, levels of oxytocin are significantly lower in those with unresolved (disorganized) attachment than in those with organized attachment patterns, although this effect was not visible when comparing those with insecure attachment styles to those with a secure attachment style [211]. These results are worth underlining since a disorganized attachment style is significantly related to traumatic experiences in childhood, especially violence [212]. However, there is also research proving different results, namely, significantly higher oxytocin levels in patients with fearful–avoidant and dismissive–avoidant attachment styles in comparison to those with secure and anxious–preoccupied attachment styles [203]. Interestingly, a study by Ebert et al., supports probable relationships between positive childhood experiences and oxytocin levels in people with BPD [200]. The authors demonstrated that in adults with BPD, memories of the “emotional warmth” displayed by parents during their childhood are positively associated with levels of oxytocin in the plasma, which was not observed in the control group [200]. To the best of our knowledge, no research on the associations between attachment and oxytocin in adolescents with borderline personality disorder has been conducted yet. However, the possible impact of disturbances in early mother–child relationships on oxytocin properties in younger groups has already been exposed by Fries et al. who discovered that in children who experienced early neglect (they were placed in orphanages directly after birth), physical contact with their adoptive mothers did not lead to a rise in oxytocin levels, while this increase was visible in children who were raised in biological families [213]. Further studies are necessary to assess thoroughly the relationship between oxytocin and attachment in people with BPD.

The potential impact of ACEs seems to be significant also in relation to the increased probability of BPD in children of parents with this diagnosis [214,215,216]. There appear to be at least two possible mechanisms behind the connection between oxytocin and the intergenerational transmission of early adverse experiences contributing to the development of borderline personality disorder [58,217].

Firstly, it has been shown that mothers with BPD manifest lower sensitivity and higher intrusiveness toward their offspring and struggle with more significant difficulties with mentalizing, also in regard to their children’s internal states [218]. In addition, mothers with this disorder show less positive affect, use vocalizing more seldom and more often exhibit ”fear/disorientation” reactions in response to infants’ needs, which, consequently, leads to worse interactions and the lower quality of their relationships both in infancy and childhood [218]. Moreover, research indicates that children of parents with borderline personality disorder are at a significantly higher risk of experiencing maltreatment than those of healthy parents [219,220]. The first possible mechanism of the involvement of oxytocin in the intergenerational transmission of ACE and borderline traits becomes more visible when we analyze these data from the perspective of how mentalization disorders in caregivers with BPD may affect the development of their children’s mentalization abilities. There exists a relationship between social cognition and oxytocin in people with borderline personality and it is fully described in the section below.

Another potential explanation of the connection between oxytocin, ACE, and borderline personality disorder is based on genetic studies. The research indicates the occurrence of an association between single nucleotide polymorphisms and the development of BPD, with the most substantial evidence pointing to *OXTR* rs53576 with the A and G alleles (AA/AG vs. GG). A study by Zhang demonstrated that in a group of male inmates in China, people with BPD showed an elevated presence of the rs53576 AA genotype compared to people without BPD, although this difference was not visible after Bonferroni correction [207]. Moreover, it has been determined that people with the GG genotype have a higher risk of developing BPD if they experience a higher intensity of physical or sexual abuse. On the other hand, with a lower intensity of these types of abuse, their risk of BPD is lower than those with AA genotype [207].

The data obtained by Cicchcetti et al. seem to confirm the hypothesis about the relationship between maltreatment in childhood, single nucleotide polymorphisms *OXTR* rs53576, and borderline traits, also in younger groups (children aged 8–12) [221]. However, its gender-dependency is speculated. The research demonstrated that among girls who experienced violence, those with the AG/AA genotype showed more borderline personality traits than girls with the GG genotype. This relationship was not visible among girls who did not experience maltreatment [221]. Interestingly, a different correlation was observed among boys—boys with the GG genotype who experienced violence showed significantly more borderline traits than those who did not experience it. Nevertheless, no differences were found in the scope of the relationships between violence and borderline traits in boys with the AG/AA genotype [221].

A study by Hammen et al. sheds a different perspective on the correlations between *OXTR* polymorphism and BPD features in adults (both genders) [222]. The relationship between the *OXTR* rs53576 single nucleotide polymorphism (AA/AG genotypes vs. the GG genotype) in interactions with intra-family conflicts as a predictor of BPD symptoms was assessed [222]. The study did not find a significant relationship between the *OXTR* genotype or family quality and BPD features when analyzed separately, yet a statistically significant association was discovered between the interaction of the *OXTR* genotype and family quality with BPD features—the effects of *OXTR* rs53576 A-polymorphism were strongly dependent on whether early relations in the family were caring and harmonious or difficult. This connection, however, was not visible for GG homozygotes. There was also no gender dependence [222]. Researchers find it reasonable to conclude that AA/AG genotype carriers are more susceptible to the quality of environmental factors [222,223]. They postulate that allelic variations which, on one hand, seem to lead to the development of maladaptive characteristics of BPD under the influence of unfavorable environmental factors may, simultaneously, produce enhanced positive effects of the same genetic factors in favorable environmental conditions [222]. The above-mentioned mechanism can provide an explanation of why borderline personality psychopathology is constantly present in the population [222].

Curiously, there is a study demonstrating an interplay between *OXTR* rs53576 gene variants, the perception of social support, and the severity of internalizing problems among maltreated 13–15-year-olds (both genders, without a BPD diagnosis). Adolescents with the GG genotype perceived less social support and experienced higher severity of internalizing problems, compared to those with the AA/AG genotypes [224]. The authors of this study indicate that one of the possible explanations for the above result is the data which suggest that people with the GG genotype are more prosocial, sensitive to social signals, and empathetic [224,225,226,227,228,229]. Those characteristics are likely to make them prone to seeking interactions with the perpetrator more frequently and to experiencing the negative mental states of their violent parents more strongly, which, in general, can make them more affected by maltreatment [224]. Although the above-described study does not regard adolescents with borderline personality disorder, the mechanism suggested by its authors seems worth considering in the light of the previously mentioned discrepant outcomes of the research on this gene.

Consequently, the data gathered to date do not allow us to formulate unambiguous conclusions. Therefore, it is necessary to examine the potential impact of different factors on the described correlations.

The above-described research becomes particularly important in the context of the previously mentioned mentalization disorders observed in people with BPD. The relationship between oxytocin, social cognition, and borderline personality disorder will be discussed in detail in the following paragraph.

### 6.3. Oxytocin and Social Cognition and Behaviors in People with Borderline Personality Disorder

#### 6.3.1. Social Cognition

Study results demonstrate possible connections between oxytocin and social cognition in people with borderline personality disorder. Notably, the available data indicate that oxytocin increases the capacity for affective empathy in people with BPD [230]. It has been shown that BPD patients tend to focus excessively on negative emotions in other people’s faces—they evince negativity bias in facial emotion recognition [58,231]. Furthermore, they are more likely to attribute anger to ambiguous facial expressions than healthy people [58,232]. Studies report that a single dose of oxytocin reduces hyperactivation of the amygdala in women with BPD, which is correlated with the normalization of attention bias in relation to negative social cues [8,233]. The above data seem to be essential concerning the previously mentioned tendency of people with borderline personality to attribute internal states to other people without objective premises for their existence, i.e., hypermentalization. The most recent study indicates the existence of a relationship between hypermentalization and reduced levels of the protein expression of oxytocin receptors in blood mononuclear cells (OXTR) in people with this disorder [234]. Importantly, no such relationship was observed in regard to plasma oxytocin levels [234].

There are insufficient data on the impact of oxytocin on social cognition in adolescents with borderline personality disorder. However, it is worth mentioning that there is some evidence suggesting potential associations in this regard. Among others, there is research on teenagers from residential youth care facilities who were admitted to these places due to externalizing problems and/or adverse family environments. The collected evidence demonstrates that the intranasal administration of oxytocin improves their ability to empathize, especially among participants with highly callous–unemotional traits [235]. These results may imply the existence of a relationship between the administration of oxytocin and empathy in adolescents with BPD if we take into account, firstly, data demonstrating associations between BPD and externalizing disorders and, secondly, the fact that diagnosing borderline personality in adolescents has only recently been more recognized as justified [236,237].

#### 6.3.2. Approach and Avoidance

The existing research on approach and avoidance in people with borderline personality disorder is also worth mentioning. There is evidence that oxytocin increases the motivation of people with BPD to approach other individuals [8,230]. The study by Schneider et al. showed that women diagnosed with borderline personality disorder responded faster to angry faces than to happy faces, in comparison with healthy adults. Moreover, healthy people responded faster to happy faces than to angry faces, compared to people with BPD [87]. Interestingly, adults with borderline personality, contrary to healthy adults, did not show the “congruence effect”—they approached happy and angry faces just as quickly. Moreover, after the administration of oxytocin, an increase in reaction times in situations of incompatibility (i.e., when approaching angry faces and staying away from happy faces) was observed, both in the group of people with BPD and the group of healthy people, compared to the study participants who received a placebo. As a result, administering oxytocin led to BPD patients reacting in a pattern typical for healthy people, i.e., it took them less time to make a decision to avoid angry faces than to approach them [87]. On the other hand, there are also some data suggesting that in people with BPD, the administration of oxytocin reduces avoidant responses to angry faces generally regarded as a form of social threat. The above-mentioned effect was not visible in healthy participants of this study [58,238].

#### 6.3.3. Stress

Studies demonstrate that in a group of patients with borderline personality, intranasal oxytocin administration attenuates negative emotional reactions and tends to reduce salivary cortisol levels in situations of social stress, compared to a placebo [58,239]. Notably, childhood trauma was the strongest predictor of the effect of oxytocin on emotional stress reactivity, expressed by stress-induced dysphoria. In addition, stress-induced cortisol increase was predicted by an insecure attachment alone and no relationship has been found in the case of childhood trauma [239].

#### 6.3.4. Trust

The existing evidence establishes that adults with borderline personality disorder, in comparison with healthy adults, tend to perceive other people’s faces as less trustworthy [58,240]. Furthermore, it has been found that, paradoxically, oxytocin reduces trust in people with BPD [241,242]. It is, however, crucial to emphasize that there may be different factors affecting those patients, such as experiencing violence in childhood or their attachment styles [8,241,242]. Bartz et al. demonstrated that oxytocin reduces trust in patients with BPD who exhibit an anxious attachment style and are sensitive to rejection, whereas, in the case of people with an anxious attachment style but with a low level of avoidance, trust is increased [241]. Moreover, Ebert et al. found a correlation between a decrease in trust after the administration of oxytocin in people with this disorder and their experience of violence in childhood [242].

The research seems to confirm a significantly lower level of interpersonal trust in adolescents with borderline personality disorder [243]. However, the relationship between oxytocin and trust in adolescents with BPD has not yet been investigated. More general data, indirectly emphasizing the noteworthiness of investigating these relationships, were found in a study that focused on assessing intranasal oxytocin effects on adolescents’ trust within an attachment-based and non-attachment-based relationship while playing a game [244]. The study included both healthy adolescents and adolescents treated in a psychiatric ward (among them, 77% met the criteria for depressive episodes, 33% for externalizing disorders, and 64% for anxiety disorders). The research task involved playing a trust game both with mothers and with strangers via the Internet [244]. The researchers demonstrated differences between patients who received a placebo and those who received oxytocin. Patients in the latter group were significantly more involved in playing the game with strangers [244]. The authors also found a significant difference between the group of patients receiving oxytocin and the group of healthy adolescents who were administered oxytocin—the former became more strongly engaged in the game than the latter [244]. Notably, there were no differences between patients who received oxytocin and healthy adolescents receiving a placebo, which confirms the researchers’ hypothesis, according to which oxytocin strengthens trust-based behavior in patients to the level of healthy people [244]. Moreover, the effect of oxytocin was visible in this group only while playing with strangers (non-attachment relationships) and not when playing with mothers (attachment-related), which implies that the oxytocin effect is weakened by an existing relationship [244]. More research is, undoubtedly, needed to assess associations between oxytocin and trust in adolescents with BPD.

#### 6.3.5. Abandonment

The research found that in adults with borderline personality disorder, after experiencing experimentally induced feelings of rejection and isolation (while playing a virtual ball-tossing game during which players excluded the study participants), the level of oxytocin in the plasma decreased, compared to people without this disorder [206,211]. Notably, it has been shown that people with borderline personality, having been socially excluded during the game, exhibit more pronounced negative emotional states [206]. Furthermore, it has been determined that there is a negative correlation between the time needed for oxytocin to return to the baseline and experiencing physical and emotional violence in childhood [206]. In the latter of the above-mentioned studies, Jobst et al. demonstrated that baseline plasma levels of oxytocin in a group of patients with borderline personality disorder and unresolved (disorganized) attachment representations were significantly lower than in patients with BPD and organized attachment representations. However, the degree to which the level of oxytocin changed after social exclusion did not differ in regard to attachment representations [211].

Regrettably, there is a lack of research addressing these issues in relation to the population of adolescents with BPD.

#### 6.3.6. Fear of Compassion

Ebert et al. reveal that adults with BPD experience higher levels of fear of compassion (FOC), which means that, not only are they more reluctant to be compassionate towards themselves and other people, but also more afraid of receiving compassion from others [200]. Compassion is associated with the motivation to care, which is essential in creating attachment. However, the authors point out that while the prime attachment is being formed, on one hand, a caregiver must be appropriately sensitive to the child’s needs, and, on the other hand, a child must also adequately respond to the parent’s attempts to calm them down and provide them with comfort [200]. At the same time, attention is drawn to the fact that children who have experienced violence tend to consider compassion as a manifestation of weakness. Moreover, they adopt a very critical attitude towards themselves as well as a belief that they do not deserve to be loved and that they are doomed to be failed by others. The above-mentioned convictions reflect the harmful and rejecting attitude of their violent caregivers towards them [245]. As a result, they exhibit both a reduced capacity to feel compassion for themselves and other people and a fear of experiencing compassion from others [245,246]. The available evidence indicates that there is a correlation between levels of FOC and levels of oxytocin in the plasma. People with BPD and a high FOC had low levels of oxytocin, whereas those with a lower FOC had higher levels of this neuropeptide [200]. Researchers postulate that this may indicate a greater role of oxytocin in the subgroup of BPD patients who have insecure attachment styles resulting from experiencing violence in early childhood [8].

The above-described relationships have not been studied in teenagers with borderline personality disorder to date.

#### 6.3.7. Aggression

The available data indicate that oxytocin levels in adults with borderline personality disorder are negatively correlated with aggressiveness [199]. Moreover, the study by Diaz-Marsá et al. demonstrated that in a group of people with BPD, lover levels of oxytocin in the plasma are marginally associated with a higher risk of verbal aggression (*p* = 0.06) and that lower OXTR expression is correlated with a higher anger trait [204].

There is a shortage of research on the associations between oxytocin and aggression in adolescents with this disorder. Indirectly, the essentiality of conducting such research has been supported by the work of Fragkaki, Cima, and Granic, who presented a model of possible associations between trauma and hormones like cortisol, testosterone, and oxytocin in adolescent aggression [7]. The authors postulate that childhood trauma leads, on one hand, to low levels of oxytocin and cortisol, but, on the other hand, to elevated levels of testosterone in people with aggressive tendencies. They argue that there is a hormonal interplay regarding trauma and aggression. According to their model, childhood trauma can affect the oxytocinergic system in males, leading to the decreased effect of oxytocin on cortisol during stress response, which, in turn, results in higher levels of cortisol. In addition, they suggested positive testosterone–cortisol coupling in adolescent boys. They propose a distinct pattern of connections between oxytocin and cortisol in adolescent girls with aggression, in which trauma affects cortisol secretion more strongly than in boys and reinforces the negative coupling between testosterone and cortisol [7].

## 7. Vasopressin and Borderline Personality Disorder

There is a paucity of research on relationships between vasopressin and borderline personality disorder. The available literature describes that people with BPD have elevated levels of vasopressin in the plasma compared to healthy people, as well as higher levels of copeptin, the c-terminal segment of the AVP precursor peptide which is considered to be a more stable substitute marker of AVP release [247].

It has been demonstrated that people with borderline personality who have augmented levels of arginine vasopressin in their plasma show increased responsiveness to negative emotions related to threats, and that the ability to recognize anger in this group is positively correlated with experiences of childhood neglect [247].

Moreover, some evidence indicates that there is a connection between the level of vasopressin in the cerebrospinal fluid and the level of aggression throughout the lives of patients with personality disorders, including borderline personality disorder [248]. However, no significant differences were found between different personality disorders. Notably, while the above-described relationship was true for both men and women, it was much stronger among males than females [248].

Although research on the relationship between vasopressin and traumatic experiences in borderline people is still lacking, there are already some data showing that trauma may affect vasopressin levels. These data, however, only apply to people with post-traumatic stress disorder. Moreover, the results of these studies do not permit us to draw unambiguous conclusions. There is evidence of reduced vasopressin levels in people with PTSD [249], yet different research shows no such associations [250].

Importantly, the role of vasopressin in adolescents with borderline personality disorder has not yet been investigated.

## 8. Factors Modulating the Action of Oxytocin and Vasopressin in People with BPD

There are various potential factors influencing the effects of oxytocin and vasopressin in patients with borderline personality disorder, including neurobiological differences (among others, genetic susceptibility), dysregulation of the oxytocin and vasopressin system as a consequence of adverse life events, social context, and, finally, numerous individual differences [8,75,247,249].

These neurohormones are speculated to be just one out of many factors affecting the development and maintenance of borderline personality disorder. Furthermore, dysfunctions in the oxytocin and vasopressin systems seem to be unspecific to BPD since they also occur in other mental disorders, including those related to attachment disturbances and experiences of violence in early childhood. The data available on this subject do not, however, enable us to draw compelling conclusions [58,251].

The possible role of conditions co-occurring in people with borderline personality disorder in generating these differences, as well as the probable impact of medications taken by patients, have also been emphasized [8].

## 9. Conclusions and Directions for Future Research

Our analysis indicates the potentially important role of oxytocin and vasopressin in the development and maintenance of symptoms of borderline personality disorder. We truly hope that all the information presented herein allows for a more comprehensive understanding of processes behind their actions in people with this condition.

A full picture of the functions of these neurohormones in the context of borderline personality disorder may lead to the creation of new diagnostic and treatment methods which, in turn, can enable clinicians to recognize this condition sooner and to obtain better treatment results. Consequently, patients may experience a decrease in the intensity of the symptoms and significant improvement in the quality of their life.

Our work demonstrates that the amount of research on this topic remains insufficient, making it impossible to draw definitive conclusions about the connections between oxytocin and vasopressin and BPD. Particular attention should be paid to the scarcity of studies on the relationship between these neuropeptides and borderline personality symptoms in the adolescent population.

The studies we analyzed are based on various methods of measuring oxytocin and vasopressin, and the vast majority of them are based on measurements of the peripheral levels of these neuropeptides, which do not allow for clear conclusions about the levels of oxytocin and vasopressin in the central nervous system. Importantly, the cited works do not always take into account the influence of other factors, which may significantly contribute to the observed divergence in the results. Moreover, it seems that further studies are necessary to fully assess differences in the distribution of levels of these neurohormones depending on age, gender, and ethnicity. Finally, the research on the population of people with BPD was carried out on potentially heterogeneous groups, including people who meet the diagnostic criteria. However, since the etiology of this disorder was not fully established, it cannot be excluded that there are different paths leading to the development of BPD, which may be manifested in a form of result discrepancies. Consequently, it is necessary to perform more studies which would consider the impact of factors such as, among others, childhood trauma.

In the context of a high incidence of borderline personality disorder among teenagers, significant clinical problems associated with BPD, as well as a lack of clearly effective treatment methods, further research in this area, which includes the above-mentioned factors and conditions, is highly recommended.

## Data Availability

Not applicable.
